# Zebrafish Krüppel-Like Factor 4a Represses Intestinal Cell Proliferation and Promotes Differentiation of Intestinal Cell Lineages

**DOI:** 10.1371/journal.pone.0020974

**Published:** 2011-06-08

**Authors:** I-Chen Li, Chein-Tso Chan, Yu-Fen Lu, Yi-Ting Wu, Yi-Chung Chen, Guo-Bin Li, Che-Yi Lin, Sheng-Ping L. Hwang

**Affiliations:** 1 Institute of Molecular and Cellular Biology, National Tsing Hua University, Hsinchu, Taiwan; 2 Institute of Cellular and Organismic Biology, Academia Sinica, Taipei, Taiwan; 3 Institute of Bioscience and Biotechnology, National Taiwan Ocean University, Keelung, Taiwan; Ecole Normale Supérieure de Lyon, France

## Abstract

**Background:**

Mouse krüppel-like factor 4 (Klf4) is a zinc finger-containing transcription factor required for terminal differentiation of goblet cells in the colon. However, studies using either *Klf4^−/−^* mice or mice with conditionally deleted *Klf4* in their gastric epithelia showed different results in the role of Klf4 in epithelial cell proliferation. We used zebrafish as a model organism to gain further understanding of the role of Klf4 in the intestinal cell proliferation and differentiation.

**Methodology/Principal Findings:**

We characterized the function of *klf4a*, a mammalian *klf4* homologue by antisense morpholino oligomer knockdown. Zebrafish Klf4a shared high amino acid similarities with human and mouse Klf4. Phylogenetic analysis grouped zebrafish Klf4a together with both human and mouse Klf4 in a branch with high bootstrap value. In zebrafish, we demonstrate that Klf4a represses intestinal cell proliferation based on results of BrdU incorporation, p-Histone 3 immunostaining, and transmission electron microscopy analyses. Decreased *PepT1* expression was detected in intestinal bulbs of 80- and 102-hours post fertilization (hpf) *klf4a* morphants. Significant reduction of alcian blue-stained goblet cell number was identified in intestines of 102- and 120-hpf *klf4a* morphants. Embryos treated with γ-secretase inhibitor showed increased *klf4a* expression in the intestine, while decreased *klf4a* expression and reduction in goblet cell number were observed in embryos injected with *Notch intracellular domain* (*NICD*) mRNA. We were able to detect recovery of goblet cell number in 102-hpf embryos that had been co-injected with both *klf4a* and *Notch 1a NICD* mRNA.

**Conclusions/Significance:**

This study provides *in vivo* evidence showing that zebrafih Klf4a is essential for the repression of intestinal cell proliferation. Zebrafish Klf4a is required for the differentiation of goblet cells and the terminal differentiation of enterocytes. Moreover, the regulation of differentiation of goblet cells in zebrafish intestine by Notch signaling at least partially mediated through Klf4a.

## Introduction

Mammalian intestinal epithelium undergoes constant proliferation, differentiation, and death. Small intestinal epithelium is composed of crypts of Lieberkuhn and villi [1]. Crypts of Lieberkuhn are pocket-like invaginations into the gut mucosa where proliferative stem cells reside to provide renewal of various differentiated intestinal cells. Differentiated intestinal cells will migrate to villi which are finger-like projections extending into the intestinal lumen. Villi are composed of three types of differentiated intestinal cells: enterocytes can absorb nutrients, goblet cells can secrete mucus as a protective barrier, and enteroendocrine cells can produce different gastrointestinal hormones to regulate growth, repair, and motility of gut epithelium. A fourth type of mature intestinal cells, Paneth cells are located at the crypt base and generate antibacterial peptides [2].

Intestinal stem cell maintenance and cell fate specification have been demonstrated to be regulated by several paracrine signaling pathways and different transcription factors [3]. Krüppel-like factors (KLFs) are evolutionary conserved zinc finger-containing transcriptional factors that regulate a variety of biological processes such as apoptosis, proliferation, differentiation, and development [4]. Among them, *klf4* (also known as *GKLF* or *EZF*) mRNA is abundantly expressed in the skin and in epithelial cells of the esophagus, stomach, and colon of newborn mice [5]. Klf4 can function as activator or repressor depending on whether the interacting protein is a co-activator such as CREB-binding protein-related protein (p300/CBP) or co-repressor such as histone deacetylase 3 (HDAC3) [6]. Due to its transactivating capability, Klf4 was shown to function in cell cycle regulation by blocking G1/S progression [7], [8]. In response to DNA damage, Klf4 mediated the transactivating effect of p53 on the *p21^WAF1/Cip1^* promoter to cause G1/S cell cycle arrest and prevented cell entry into mitosis by repressing *cyclin B1* promoter activity [9], [10]. Reduced *klf4* expression was detected in a number of colorectal cancer cell lines which was attributed to either hemizygous deletion of *klf4* gene, hypermethylation of 5′untranslated region or point mutation in the open-reading frame [11]. Decreased *klf4* expression was also identified in intestinal adenomas of multiple intestinal neoplasia mice and in colonic adenomas of familial adenomatous polyposis patients [12]. Furthermore, overexpression of *klf4* in the human RKO colon cancer cell lines resulted in reduced tumorigenecity, suggesting *klf4* is a tumor suppressor gene in colorectal cancer [13]. However, *klf4* was also found to be overexpressed in squamous cell carcinoma and was implicated to be potent oncogene [14]. Subsequent studies indicated that Klf4 can switch from a tumor suppressor to an oncogene, depending on the level of p21 in the cell [15], [16].

In addition to regulating cell proliferation, Klf4 was shown to modulate the differentiation of various tissues including gastrointestinal tract. Klf4 was shown to regulate expressions of enterocyte differentiation marker gene *intestinal alkaline phosphatase* and *zip4* gene encoding for zinc transporter [17], [18]. In *klf4^−/−^* mice, significant decrease in the number of goblet cells in the colon was detected on postnatal day1 [19], indicating Klf4 is required for terminal differentiation of goblet cells in the colon. However, other cell types including colonocytes and enteroendocrine cells, which undergo normal maturation and normal cell proliferation was detected in the colon of *klf4^−/−^* mice. In contrast, conditional deletion of *klf4* gene caused aberrant expression of acidic mucins and TFF2/SP-positive cells marked as premalignant gastric cancer and increased cell proliferation in adult stomach [20].

Notch and Wnt signaling have been implicated in the stem cell regulation and the differentiation of intestinal epithelium [3]. Usingγ-secrtase inhibitor and promoter assay, it was shown that *klf4* expression is inhibited by Notch signaling which controls goblet cell differentiation in mouse gastrointestinal tract [21]. Many colorectal cancers occur under the condition that the presence of mutation in the Wnt pathway and down-regulation of Klf4. A cross talk between Klf4 and β-catenin was established that Klf4 can bind to C terminus of β-catenin to repress its transcription [22]. Therefore, the inhibition of Wnt/β-catenin signaling by Klf4 plays an important role in the regulation of normal intestinal homeostasis and tumor repression.

Recently, zebrafish has been used as a valuable model organism to study gastrointestinal development and related human diseases [23]. Compared to mammalian intestinal epithelium, zebrafish does not contain crypts of Lieberkuhn and Paneth cells and their villi contain only three types of differentiated cells including enterocytes, goblet and enteroendocrine cells [24]. Although, a previously identified zebrafish klf4, a homologue of *Xenopus* KLF, was shown to be essential for pre-poster differentiation and hatching [25], the existence of mammalian Klf4 homologue in zebrafish embryos is still unclear. In this study, we cloned the zebrafish *klf4* homologue (*klf4a*). Both antisense morpholino oligomer knockdown and overexpression approaches were used to investigate its function in intestinal tract development. *klf4a* morphants showed increased intestinal cell proliferation while decreased intestinal cell proliferation was observed in *klf4a*-ectopically expressed embryos. These results suggest Klf4a functions as a tumor suppressor in the intestine. Decreased *PepT1* expression and significant reduction in goblet cell number were detected in *klf4a* morphants, indicating Klf4a regulates the differentiation of goblet cell and the terminal differentiation of enterocytes. Furthermore, our results demonstrate that *klf4a* expression is negatively regulated by Notch signaling and Klf4a acts as a mediator in the regulation of differentiation of goblet cells in zebrafish intestine by Notch signaling.

## Materials and Methods

### Zebrafish Maintenance and Staging

Wild type AB strain zebrafish were maintained and different developmental stages were determined as described [26], [27]. All animal procedures were approved by the Animal Use and Care Committee of Academia Sinica (protocol # RFiZOOHS2003151).

### Cloning, Plasmid Construction, Phylogenetic, and Syntenic Comparison Analyses

A pair of specific primers was designed based on *klf4a* EST sequence to obtain a 700 bp RT-PCR product as a probe to screen aλgt10 zebrafish cDNA library (Clontech). DNA and deduced amino acid sequences were analyzed using Lasergene software (DNAstar) and are deposited in GenBank under Accession no. DQ679226.

For construction of the expression vector, the *klf4a* coding region was amplified using Pfu DNA polymerase (Strategene) and cloned into a T7TS vector for capped *klf4a* mRNA synthesis. To construct Klf4a-T7 expression vector, 5′UTR and *klf4a* coding region were amplified by PCR and cloned into pCS2^+^-C-terminal 3× T7 vector [28]. Phylogenetic analyses were performed as described [26]. BioMart data mining program from the Ensembl Genome Browser was used to construct syntenic comparison analyses among zebrafish linkage group 21, human chromosome 9 and mouse chromosome 4.

### Antisense Morpholino Oligonucleotide-Mediated Knockdown Analysis

Two translational morpholino oligonucleotides (MOs) (Gene Tools) were designed to inhibit *klf4a* protein synthesis. The morpholino sequences were as follows: klf4a-MO1: CATGAGTGGAAGGAACGCAAAAG; klf4a-MO2: CAAACTCAGT CGGAGGCTGCCTCAT . Two control MOs were designed. They are Klf4a-5mmMO1: CATGAcTGcAAGcAACcgAAAAG, and Klf4a-5mmMO2: CAAA gTCAcTCGcAGGCTGgCTgAT . Diluted different MOs were microinjected into the cytoplasm of 1–2 cell zygotes using a Nanoject II automatic injector (Drummond).

### Overexpression, Recue, Notch Signaling Modulation and Western Blot Analysis

Respective capped *klf4a*, *lacZ*, and *Notch 1a intracellular domain* (*NICD*) mRNAs were synthesized using either a T7 or SP6 mMESSAGE mMACHINE kit (Ambion). To ectopically express *klf4a*, *klf4a* (150 pg) mRNA was injected into 1–2-cell zygotes, and *lacZ* mRNA (250 pg) was injected for comparison. To rescue *klf4a* morphants, *klf4a* mRNA (100 pg) was co-injected with klf4a-MO1 and klf4a-MO2 (1.5 ng each) into 1–2-cell zygotes. Co-injection of *lacZ* mRNA (100 pg) with klf4a-MO1 and klf4a-MO2 was used as control. To inhibit Notch signaling, 30-hour post fertilization (hpf) embryos were incubated with 100 μM N-[N-(3,5-Difluorophenacetyl- L-alanyl)]-S-phenylglycine *t*-Butyl Ester (DAPT) (Calbiochem) in the dark until they were fixed at 72-hpf [29]. Embryos treated with 1% DMSO were used as control. To activate Notch signaling, 200 pg *Notch 1a NICD* mRNA was injected into 1-cell zygotes while 400 pg *lacZ* mRNA was injected as control. To investigate whether klf4a is a mediator of Notch signaling on goblet cell differentiation, 100 pg *klf4a* and 300 pg *Notch 1a NICD* mRNA were co-injected into 1-cell zygotes and co-injection of 100 pg *lacZ* mRNA was performed for comparison.

Polyclonal antibody against Klf4a was generated by immunizing rabbit with His-tagged Klf4a protein without the zinc finger DNA binding domain (Qiagen) and purified by antigen-specific affinity purification using SulfoLink Protein kit (Thermo Scientific).

Total protein isolated from 72-hpf embryos that had been injected with either pCS2^+^-Klf4a-T7 plasmid DNA (100 pg), pCS2^+^-Klf4a-T7 plasmid DNA (100 pg) and klf4a-5mmMO2 (4 ng), or pCS2^+^-Klf4a-T7 plasmid DNA (100 pg) and klf4a-MO1 and klf4a-MO2 (1.5 ng each) were separated by 12% SDS polyacrylamide gel electrophoresis. Expression of Klf4a-T7 fusion protein was detected using affinity purified Klf4a antiserum (1∶100) and peroxidase-conjugated goat anti-rabbit-IgG (1∶2000; Jackson) as well as using anti-T7 antiserum (1∶5000; Calbiochem) and peroxidase-conjugated goat anti-mouse IgG (1∶5000; Jackson). Expressions of respective α-tubulin and proliferating cell nuclear antigen (PCNA) proteins were detected using either anti-α-tubulin antiserum (1∶1000; Sigma) or anti-PCNA antiserum (1∶1000; Caltag). Peroxidase-conjugated goat anti-mouse IgG (1∶10,000; Sigma) was used to detect expression of both α-tubulin and PCNA proteins. ECL Plus Western Blotting Detection system (Amersham) was used to visualized protein band on Blue XB-1 film (Kodak).

### Whole-Mount In Situ Hybridization, Alcian Blue Staining, BrdU Incorporation and p-Histone H3 Immunostaining

Whole-mount *in situ* hybridization was conducted on embryos treated with 0.003% phenylthiocarbamide using digoxigenin-labeled antisense RNA probes and alkaline phosphatase-conjugated anti-digoxigenin antibodies as described [30]. Various templates were linearized, and antisense RNA probes were produced as follows: *her6* (*Bam* HI/T7); *glucagon* (*Nco* I/SP6); *klf4a* (*Hind* III/T3); *PepT1* (*Nco* I/SP6).

Whole-mount alcian blue staining on *klf4a* morphants and control embryos were conducted as described [31]. 5-bromo-2-deoxyuridine (BrdU) labeling and p-Histone H3 immunostaining on *klf4a* morphants and control embryos were conducted as described [31]. In order to evaluate the differentiation of enteroendocrine and goblet cell at the same time, *glucagon*- hybridized embryos were embedded for paraffin sectioning. After wax removal and rehydration, these sections were rinsed with acid alcohol and incubated with 0.1% alcian blue 8GX in acid alcohol for 30 min [31]. Finally, they were stained with hematoxylin according to standard procedures.

### Histology, Photography, and Electron Microscopy

Paraffin sectioning, hematoxylin and eosin staining were conducted based on standard procedures. Images of embryos were taken using an RT color digital camera (SPOT) on a Zeiss Axioplan 2 microscope equipped with DIC mode. The obtained images were used to count goblet and enteroendocrine cell numbers and p-Histone H3-and BrdU-labeled cell percentages in the intestine manually. Area of goblet cells was calculated using AixoVision software (Zeiss). BrdU-labeled cell percentages for different parts of intestine were calculated as described [31]. Percentages of p-Histone H3-labeled cells, *glucagon*-expressing enteroendocrine cells and alcan blue-stained goblet cells were respectively calculated by counting labeled cell number and the total intestinal cell numbers in those longitudinal sections containing complete intestines of *klf4a* morphants, *klf4a*-injected, *lacZ*-injected, and wild type embryos. Transmission electron microscopy analysis was performed as described previously [31].

### Total RNA Extraction and Real-Time Quantitative Reverse-Transcription –PCR (qPCR)

Total RNA was extracted using RNeasy Plant Mini Kit (Qiagen). To quantify expression levels of *PepT1*, *klf4a*, and *β-actin*, qPCR was conducted as described [31]. *klf4a* primer pair was F-TCGGAACCGAACATGCTGCTTTG and R-CGCTCAGTGCCATGCTATCAAAC. The primer pair for *PepT1* and *β-actin* were the same as described [31].

### DNA Microarray and Gene Ontology (GO) Analyses

Total RNA of 96-hpf wild type and *klf4a* morphant embryos were extracted with RNeasy Plant Mini Kit (Qiagen) and their qualities were verified by Bioanalyzer 2100 (Agilent). Respective 10 µg total RNA was converted to double-stranded cDNA using SuperScript Double-Stranded cDNA Synthesis Kit (Invitrogen). Before precipitation for overnight at −20°C, the synthesized double-stranded cDNA samples were incubated with RNase A at 37°C for 10 min. The pellets were rehydrated with nuclease-free water and their qualities were confirmed by Bioanalyzer 2100. Respective 1 µg cDNA was labeled with Cy3-Random Nonamers using One-Color DNA Labeling Kit (NimbleGen) and then hybridized with Zebrafish Gene Expression 385K Arrays (NimbleGen) that containing 38,489 genes with designed 12 probes of 60 mer oligonucleotides per gene at 42°C for 18 h. The slide was washed in Wash I, II and III (NimbleGen) and then scanned with GenePix 4000B Microarray Scanner (Molecular Devices) after it was dried. A list of up- and down-regulated genes was uploaded to the website of Gene Ontology Enrichment Analysis Software Toolkit (GOEAST, http://omicslab.genetics.ac.cn/GOEAST/tools.php) and analyzed through Batch-Genes program using database of *Danio rerio* with p-value smaller than 0.1 [32].

## Results

### Cloning of the Zebrafish *klf4a* Gene, Phylogenetic, and Syntenic Comparison Analyses

Full-length *klf4a* cDNA was obtained by screening a zebrafishλphage cDNA library. The deduced amino acid sequence showed that zebrafish *klf4a* encodes a 396-amino acid-long polypeptide. Amino acid sequence comparison among human, mouse Klf4 and zebrafish Klf4a revealed the presence of 3 conserved C-terminal zinc finger DNA binding domains, preceded nuclear localization signal, and the N-terminal activation domain containing acidic residues (Figure S1A). Zebrafish Klf4a shared 66–67% amino acid similarities with human and mouse Klf4. Phylogenic analysis demonstrated that zebrafish Klf4a is grouped together with human and mouse Klf4 with a high bootstrap value, while a previously identified zebrafish Klf4 was branched together with Klf2 members from mouse and zebrafish (Figure S1B). Syntenic analyses were conducted to compare conservation of genes, including *Klf4* homologues, among zebrafish linkage group 21, human chromosome 9, and mouse chromosome 4 (Figure S2). We identified 10 out of 14 genes from zebrafish linkage group 21 sharing equal or greater than 50% amino acid sequence identity with respective homologue in both human chromosome 9 and mouse chromosome 4. However, the orders of loci within the chromosome are quite rearranged. These results indicate that zebrafish Klf4a is the homologue of mammalian Klf4.

### Expression Patterns of *klf4a* and Its Expression was Inhibited by Notch Signaling

**Figure 1:**
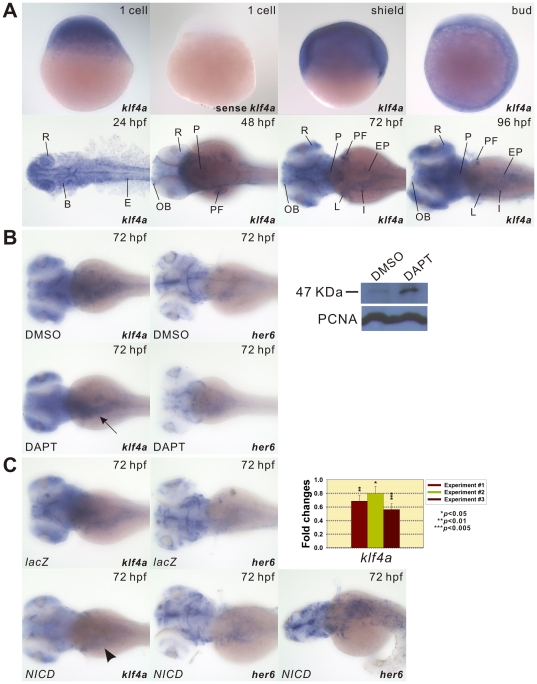
Developmental expression patterns of *klf4a* and the effect of Notch signaling on *klf4a* expression. (A) Embryos from different developmental stages were hybridized with either antisense *klf4a* or sense *klf4a* RNA probes as indicated. Lateral view is shown for 1 cell, shield, and bud embryos. Dorsal view is shown for 24–96-hpf embryos. B, brain; E, epidermis; EP, exocrine pancreas; I, intestine, L, liver; OB, olfactory bulbs; P, pharynx; PF, pectoral fin; R, retina. (B) Enhanced *klf4a* expression in intestines (arrow) of DAPT-treated embryos is shown by *in situ* hybridization and Western blot analyses. Decreased *her6* expression in the brain and intestines of DAPT-treated embryos is shown by *in situ* hybridization. Similar PCNA expression levels are shown in DMSO- and DAPT-treated embryos. (C) Decreased *klf4a* expression in intestines (arrowhead) of *NICD*-overexpressed embryos is shown by *in situ* hybridization and qPCR. Increased *her6* expression in the brain and intestines of *NICD*-overexpressed embryos is shown by *in situ* hybridization. Error bars indicate the standard error. Student's *t*-test was conducted to compare *lacZ*- and *NICD*-ectopically expressed embryos.

Zebrafish *klf4a* mRNA is a maternal transcript as shown by hybridization signal in 1-cell zygotes (Figure 1A). Ubiquitous *klf4a* expression was detected in both shield and bud embryos. At 24-hpf, *klf4a* was expressed in the retina, ventral brain, and epidermis of the whole embryos. *klf4a* mRNA was localized in the olfactory bulbs, retina, pharynx, pectoral fin buds, and epidermis of 48-hpf embryos (Figure 1A and Figure S3). At 72-hpf, *klf4a* was expressed in the olfactory bulbs, retina, left and right habenula neurons, cranial neurons, pharynx, pectoral fin, pharyngeal arches, liver, intestinal bulb and exocrine pancreas (Figure 1A and Figure S3). At 96-hpf, *klf4a* mRNA was distributed in similar tissues as those observed in 72-hpf embryos except that *klf4a* was expressed in the entire intestine (Figure 1A and Figure S3).

Since Notch signaling has been shown to inhibit mouse *klf4* expression [21], we also investigated whether similar regulatory mechanisms exist in zebrafish embryos. Increased *klf4a* expression in the intestine was detected in 72-hpf DAPT-treated embryos as compared to DMSO-treated embryos (Figure 1B). Decreased expression of *her6*, a downstream target of Notch, was identified in DAPT-treated embryos by whole-mount *in situ* hybridization, demonstrating the effectiveness of DAPT treatment. In order to ensure the specificity of whole-mount *in situ* hybridization, we raised polyclonal antibody by immunizing rabbit with His-tagged Klf4a protein without the zinc finger DNA binding domain. A faint 47 kDa protein band corresponding to expected size of endogenous Klf4a protein can be recognized by Klf4a antiserum in the lane loaded with total protein isolated from 72-hpf DMSO-treated embryos. In contrast, a prominent 47 kDa protein band was recognized by Klf4a antiserum in the lane loaded with total protein obtained from DAPT-treated embryos (Figure 1B).

In contrast, reduced *klf4a* expression was detected in intestines of 72-hpf *NICD*-ectopically expressed embryos as compared to embryos that had been injected with 400 pg of *lacZ* mRNA (Figure 1C). Increased expression of *her6* was identified in *NICD*-ectopically expressed embryos, demonstrating the effectiveness of *NICD* overexpression. qPCR further confirmed significant decrease of *klf4a* mRNA levels. The similarity in the regulation of zebrafish *klf4a* and mammalian homologue, *klf4*, by the Notch signaling, further support the notion that they are homologues.

### Antisense MO-Mediated Knockdown of *klf4a* Expression

Two klf4a specific translational antisense MOs (klf4a-MO1 and klf4a-MO2) were designed to study *Klf4a* function by inhibiting its protein synthesis. Respective five mismatches was introduced in these two klf4a-MOs and used as controls in this study. Initial experiments, conducted to determine optimal doses needed, indicated that it required injecting either 4 ng klf4a-MO1 or 16 ng klf4a-MO2 to achieve high (92%; 62%) percentage morphant rate and morphant phenotype (Table S1 and Figure S4). However, co-injection of 1.5 ng each of klf4a-MO1 and klf4a-MO2 can obtain the highest (98%) percentage morphant rate and morphant phenotype. These results indicate that there is a synergistic effect between these two translational klf4a-MOs, and co-injection of 1.5 ng each of klf4a-MO1 and klf4a-MO2 was used to generate *klf4a* morphants in the following experiments.

**Figure 2:**
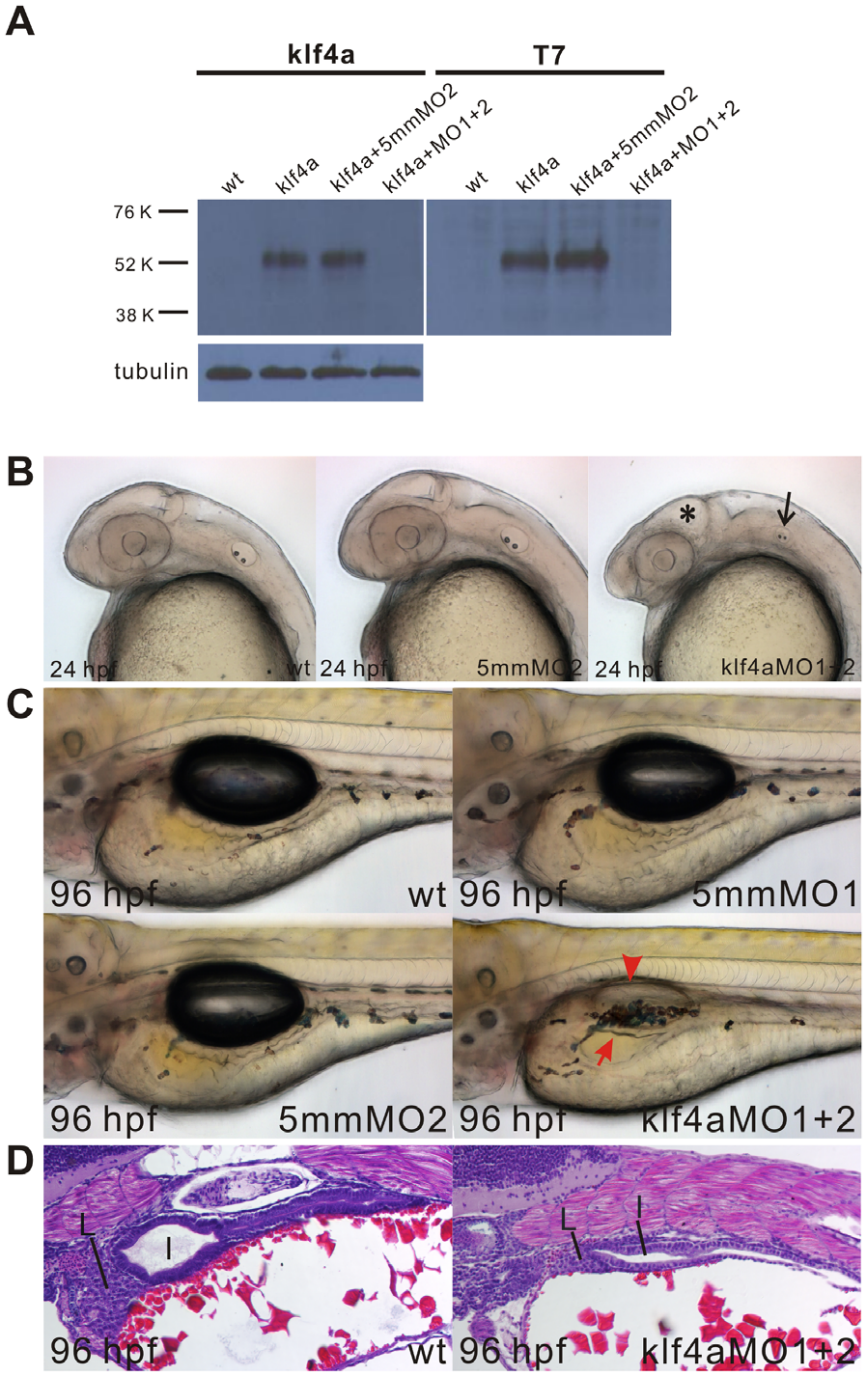
Phenotype and histological analysis of klf4a-MO1 and klf4a-MO2-injected morphants and the specificity of klf4a-MOs by Western blot analyses. (A) Western blot analyses demonstrate that co-injection of klf4a-MO1 and klf4a-MO2 (1.5 ng each) prevents the synthesis of klf4a-T7 fusion protein. Similar α-tubulin expression levels are shown in embryos with different treatments. (B) Phenotype comparison among wild type, klf4a-5mmMO2-injected, and klf4a-MO1 and klf4a-MO2-injected embryos at 24 hpf. * indicates the enlargement of midbrain ventricle and the arrow indicates the reduced otic vesicle. (C) Intestinal phenotype comparison among wild type, klf4a-5mmMO1-, klf4a-5mmMO2, and klf4a-MO1 and klf4a-MO2-injected embryos at 96 hpf. The arrow indicates absence of intestinal folding and the arrowhead indicates no inflation of swim bladder. (D) Histological analysis of 96-hpf klf4a-MO1 and klf4a-MO2-injected morphants. I, intestine; L, liver.

To test the effectiveness of MOs inhibiting the expression of *klf4a*, we carried out western blot analysis. Since endogenous Klf4a protein level is low, we overexpressed *klf4a* by injecting a pCS2^+^-Klf4a-T7 plasmid DNA into 1–2 cell zygotes and detected a 52 kDa protein band corresponding to the expected size of Klf4a-T7 fusion protein from 72-hpf injected embryos (Figure 2A). Synthesis of the 52 kDa Klf4a-T7 fusion protein was inhibited in embryos that had been co-injected with pCS2^+^-Klf4a-T7 plasmid DNA and klf4a-MO1 and klf4a-MO2 while the 52 kDa Klf4a-T7 fusion protein still can be detected in embryos that had been co-injected with pCS2^+^-Klf4a-T7 plasmid DNA and klf4a-5mmMO2. These results demonstrated the specificity of two translational klf4a MOs used in this study.

Notable morphological changes such as smaller eyes and otic vesicles, reduced dorsal-ventral axis, and the enlargement of midbrain ventricle were detected in 24-hpf *klf4a* morphants as compared to wild type and embryos that had been injected with respective 4 ng klf4a-5mmMO1 or klf4a-5mmMO2 (Figure 2B and Figure S4). In 96-hpf *klf4a* morphants, no intestinal folding and no inflation of swim bladder could be detected (Figure 2C and Figure S4). Histological analyses further demonstrated hypoplastic development of liver, intestines and exocrine pancreas in 96-hpf *klf4a* morphants (Figure 2D and data not shown).

### Decreased *PepT1* Expression Detected in Enterocytes of Intestines of *klf4a* Morphants

**Figure 3:**
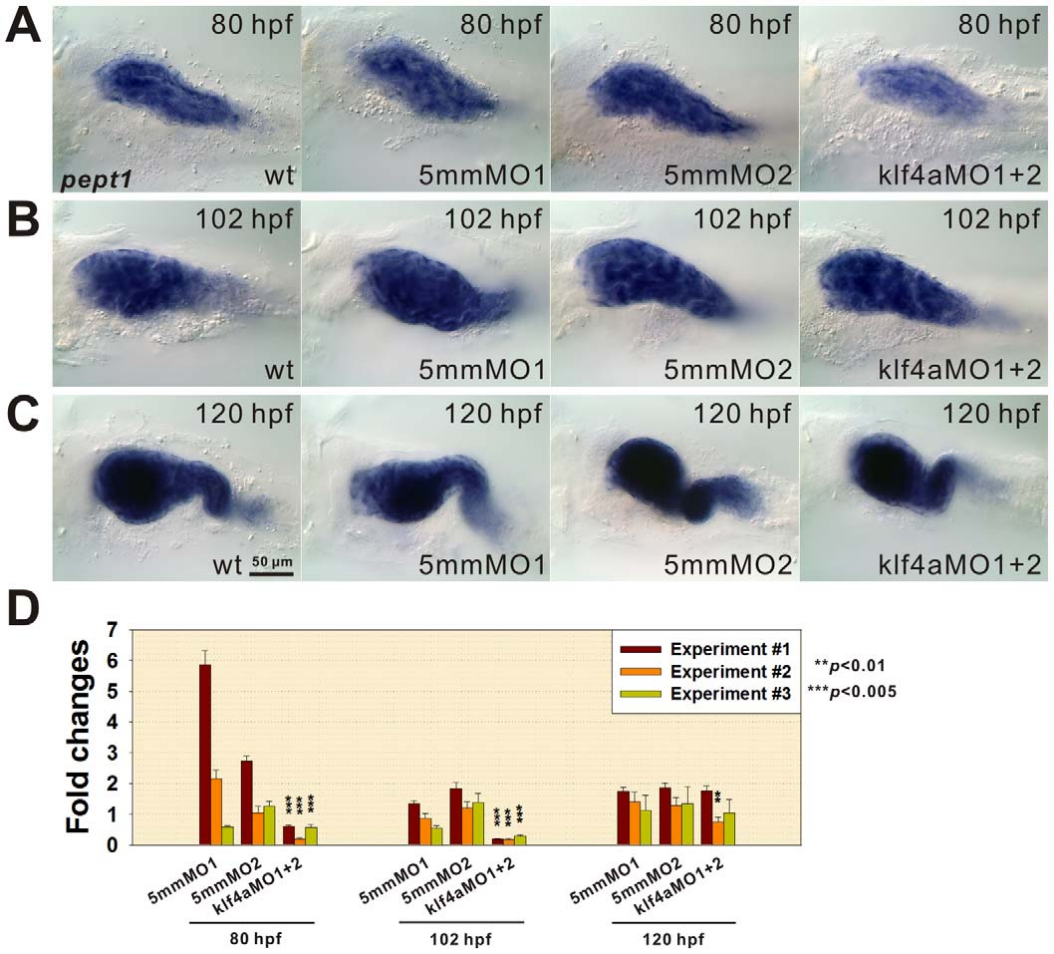
Decreased *PepT1* expression is detected in *klf4a* morphants. *PepT1* expression comparison among 80- (A), 102- (B), and 120-hpf (C) klf4a-MO1 and klf4a-MO2-injected morphants, klf4a-5mmMO1-, klf4a-5mmMO2-injected, and wild type embryos. (D) *PepT1* expression level analyzed by qPCR. Error bars indicate the standard error. Student's *t*-test was conducted to compare *klf4a* morphants with either wild type, klf4a-5mmMO1- or klf4a-5mmMO2-injected embryos.

Zebrafish intestines contain three types of differentiated epithelial cells including enterocytes, enteroendocrine and goblet cells. The oligopeptide transporter, PepT1 is considered as a terminal differentiation marker of enterocytes [33]. The expression intensity of *PepT1* in the intestinal bulb of 80-hpf *klf4a* morphants was decreased substantially when compared to control embryos (Figure 3). While *PepT1* expression levels in 102- and 120-hpf *klf4a* morphants were not substantially decreased as compared to respective control embryos, however qPCR showed significant decreased *PepT1* mRNA levels in both 80- and 102-hpf but not in 120-hpf *klf4a* morphants. These data indicates that Klf4a regulates enterocyte terminal differentiation.

### Significant Decreased Goblet Cell Number Detected in Intestines of *klf4a* Morphants

**Figure 4:**
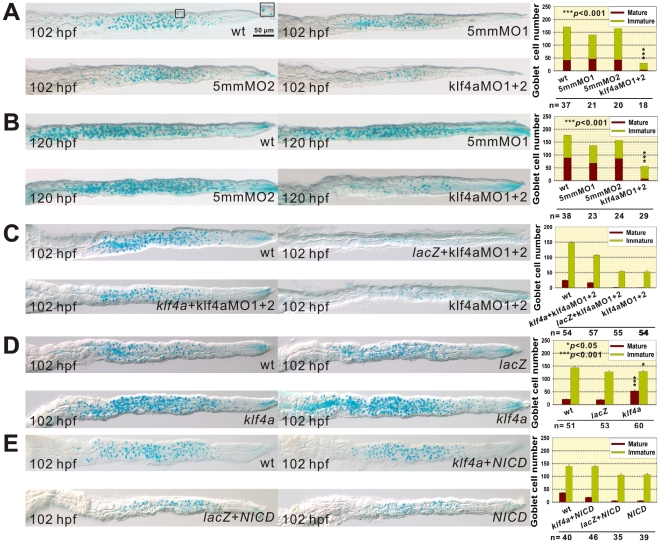
Altered goblet cell numbers are detected in *klf4a* morphants, *klf4a*-overexpressed, and *NICD*-overexpressed embryos. Decreased goblet cell number is observed in 102- (A) and 120-hpf (B) klf4a-MO1 and klf4a-MO2-injected morphants as compared with klf4a-5mmMO1-, klf4a-5mmMO2-injected, and wild type embryos by alcian blue staining and goblet cell number quantification. Inlet figure shows a mature and an immature goblet cell. Error bars indicate the standard error. Student's *t*-test was conducted to compare both immature and mature goblet cell numbers in *klf4a* morphants with those in either wild type, klf4a-5mmMO1- or klf4a-5mmMO2-injected embryos. (C) Co-injection *klf4a* mRNA with klf4a-MO1 and klf4a-MO2 can rescue both mature and immature goblet cell numbers by alcian blue staining and goblet cell number quantification. (D) *klf4a* overexpression increases goblet cell number by alcian blue staining and goblet cell number quantification. Error bars indicate the standard error. Student's *t*-test was conducted to compare both mature and immature goblet cell numbers in *klf4a*-overexpressed embryos with those of either wild type or *lacZ*-overexpressed embryos. *p<0.05 exists only for the comparison of immature goblet cell number in *klf4a*-overexpressed and wild type embryos. (E) Co-injection *klf4a* and *Notch1a NICD* mRNAs can rescue both mature and immature goblet cell numbers by alcian blue staining and goblet cell number quantification.

To investigate role of *Klf4a* in the differentiation of goblet cells, we used alcian blue staining to detect sulfated and carboxylated sialomucins in intestinal goblet cells. Since the differentiation of goblet cells involved the initial formation of pre-goblet cells containing few mucous granules to become mature goblet cells which contains numerous mucous granules, we defined those alcian blue-stained goblet cells with area equal or larger than 24.0±0.5 μm^2^ (*n* = 86) as mature goblet cells and those with area less than this value as immature goblet cells. We observed 90% (*n^102^* = 18; *n^120^* = 29) reduction of mature goblet cell number and respective 80% to 46% decrease of immature goblet cell number in 102- and 120-hpf *klf4a* morphants as compared with control embryos (Figure 4A and 4B).

In order to demonstrate the differentiation defect of goblet cell is due to the absence of Klf4a protein, we conducted rescue experiment. Co-injection *klf4a* mRNA with both klf4a-MO1 and klf4a-MO2 can rescue 13.7% (*n* = 57) of mature goblet cell number in 102-hpf injected embryos which is comparable to 14.3% (*n* = 54) mature goblet cell number detected in wild type embryos. In contrast, co-injection *lacZ* mRNA (*n* = 55) did not have any effect (Figure 4C). Furthermore, we conducted *klf4a* overexpression experiment and discovered that a 2.8 fold (*n* = 60) increase of mature goblet cell number was detected in 102-hpf embryos that had been injected with *klf4a* mRNA but not with *lacZ* mRNA (*n* = 53) (Figure 4D). These results demonstrate that decreased goblet cell number observed in *klf4a* morphants is specifically due to deficiency of Klf4a protein and *klf4a* expression is important for the differentiation of goblet cells in intestines of zebrafish embryos.

A previous study was shown that Delta-Notch signaling controls the intestinal secretory cell fate commitment in zebrafish, and intestinal cells almost adopted secretory cell fate when Delta-Notch signaling was blocked [34]. We detected reduction in both immature and mature (6.7 fold decrease; *n* = 39) goblet cell numbers in *NICD*-ectopically expressed 102-hpf embryos as compared to wild type embryos (Figure 4E). However, adequate recovery of both immature and mature (11.9%, *n* = 46) goblet cell numbers was identified in 102-hpf embryos that had been co-injected with both *klf4a* and *Notch 1a NICD* mRNA as compared to wild type embryos (*n* = 40) that containing 20.9% mature goblet cells. While, no rescue effect can be observed in embryos that had been co-injected with both *lacZ* and *Notch 1a NICD* mRNA (*n* = 35). These results indicate that the regulation of differentiation of goblet cells in zebrafish intestine by Notch signaling at least partially mediated through Klf4a.

### Decreased *glucagon*-Expressing Enteroendocrine Cell Number Detected in Intestines of *klf4a* Morphants

**Figure 5:**
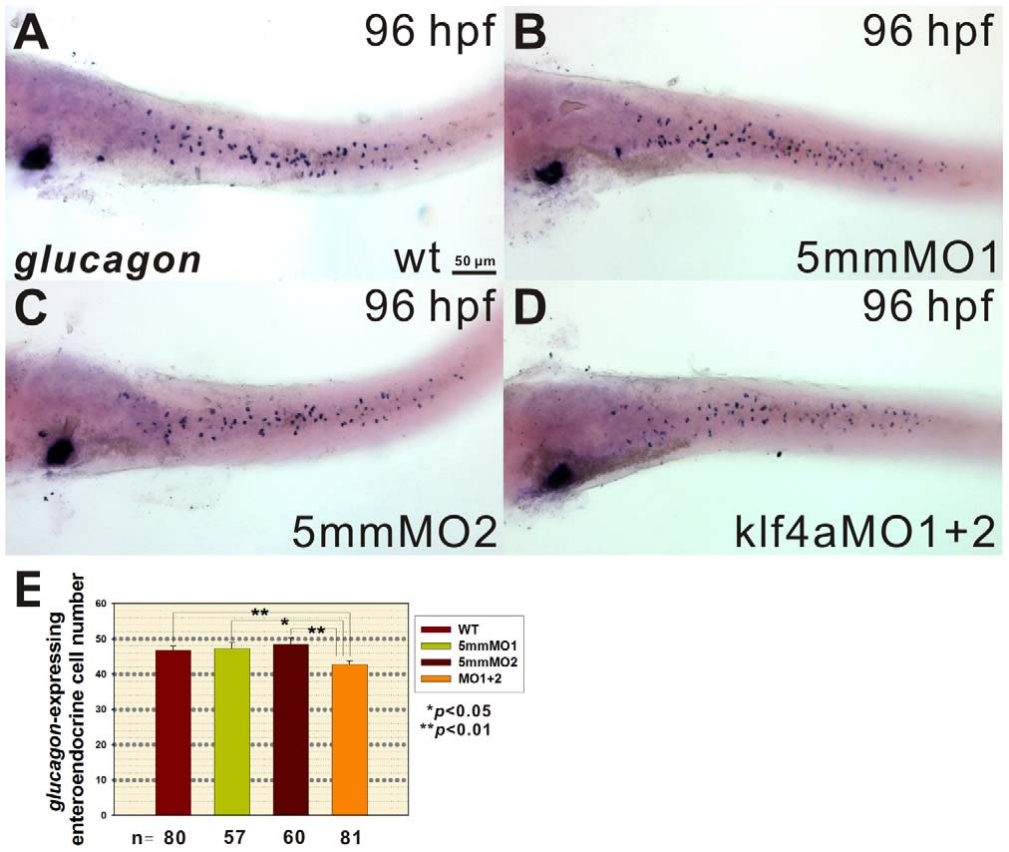
Reduction in *glucagon*-expressing enteroendocrine cell number is detected in *klf4a* morphants. *glucagon* RNA probe is used in whole-mount *in situ* hybridization to detect enteroendocrine cells in 96-hpf (A) wild type, (B) klf4a-5mmMO1- (C) klf4a-5mmMO2-injected, and (D) klf4a-MO1 and klf4a-MO2-injected embryos. (E) Quantification of *glucagon*-expressing enteroendocrine cell number in 96-hpf embryos with different treatments. Error bars indicate the standard error. Student's *t*-test was conducted to compare *klf4a* morphants with either wild type, klf4a-5mmMO1-, or klf4a-5mmMO2-injected embryos.

Intestinal enteroendocrine cells are another type of secretory intestinal cells, they produce different hormones, including glucagon, which is also synthesized in pancreatic α cells. A small but significant decreased numbers of *glucagon*-expressing enteroendocrine cells were detected in the posterior intestinal bulb and mid-intestine of 96-hpf *klf4a* morphants as compared to control embryos (Figure 5). These results suggest that *klf4a* expression is also somewhat important for the differentiation of enteroendocrine cells in intestines of zebrafish embryos.

### Analyses of Secretory Cell Differentiation along Anterior-Posterior Axis of Intestines in *klf4a* Morphants

**Figure 6:**
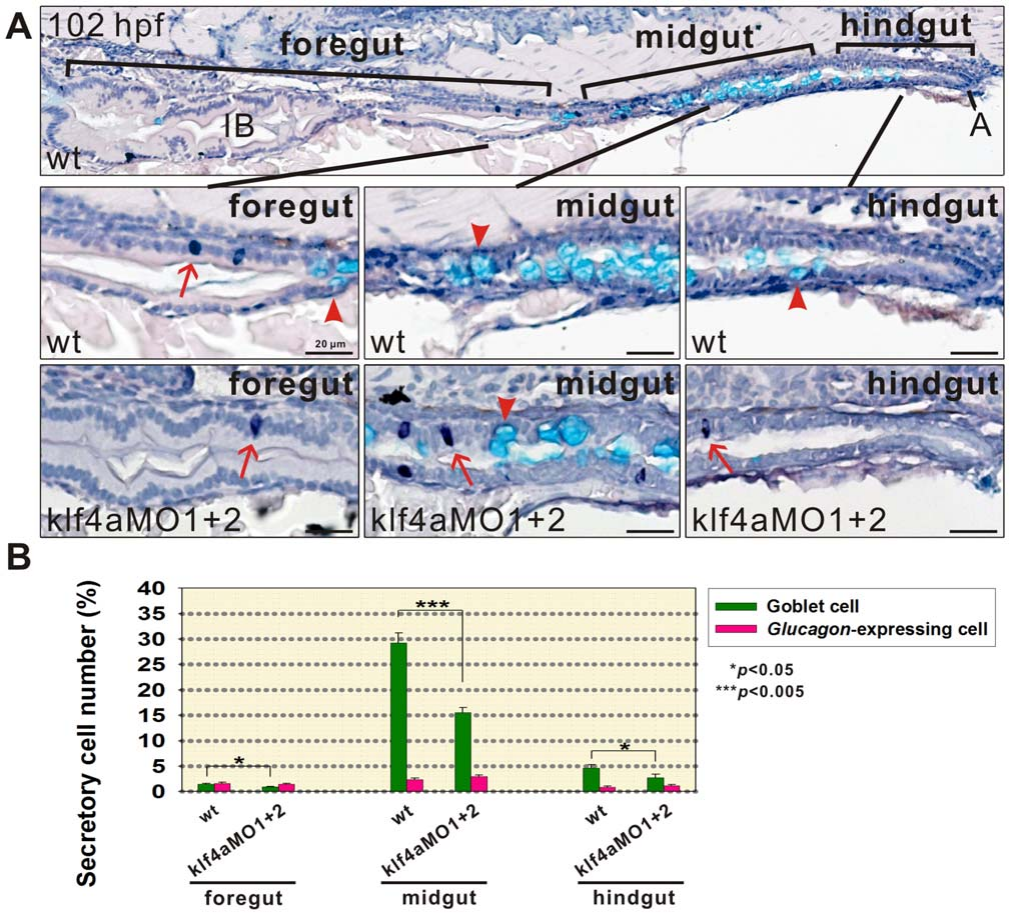
Analysis of secretory cell differentiation along anterior-posterior axis of intestines in *klf4a* morphants. 102-hpf wild type and *klf4a* morphant embryos were hybridized with *glucagon* RNA probe and processed for paraffin embedding and sectioning. Paraffin sections were then used for alcain blue and hematoxylin staining. (A) A section shows complete intestine including intestinal bulb (IB)/foregut, mid-intestine/midgut and posterior intestine/hindgut of a wild type embryo. Corresponding enlarged images of foregut, midgut, and hindgut of both wild type and klf4a-MO1 and klf4a-MO2-injected embryos are shown. Arrows indicate *glucagon*-expressing enteroendocrine cells and arrowheads indicate alcian blue-stained goblet cells. (B) Comparison of the percentages of goblet cell and *glucagon*-expressing enteroendocrine cell numbers within different segments of intestines in 102-hpf wild type and klf4a-MO1 and klf4a-MO2-injected embryos. Error bars indicate the standard error. Student's *t*-test was conducted to compare *klf4a* morphants with wild type embryos. A, anus; IB, intestinal bulb.

To further investigate the effect of KLF4a on the differentiation of two secretory cells along anterior-posterior axis of intestines, 102-hpf wild type and *klf4a* morphant embryos were first hybridized with *glucagon* RNA probes to label *glucagon*-expressing enteroendocrine cells by whole-mount *in situ* hybridization. Respective *glucagon*-hybridized embryos were then processed for paraffin embedding and sectioning. Paraffin sections were used for alcian blue staining to label goblet cells and subsequent hematoxylin staining were used to identify all intestinal cells including enterocytes. Cell numbers of alcian blue-stained goblet cells, *glucagon*-expressing enteroendocrine cells and the rest of enterocytes were quantified from a number of longitudinal sections containing complete intestines along anterior to posterior axis. As shown in Figure 6, *glucagon*-expressing enteroendocrine cells were scattered from distal intestinal bulb/foregut to posterior intestine/hindgut while goblet cells were mainly distributed in the mid-intestine/midgut. The distribution of *glucagon*-expressing enteroendocrine cells remained to be similar while the distribution of goblet cells was altered in 102-hpf *klf4a* morphants (*n* = 10) as compared with wild type embryos (*n* = 7) (Figure 6A). Significant decreases in the number of goblet cells in *klf4a* morphants within intestinal bulb (1.6 fold), mid-intestine (1.9 fold), and posterior intestine (1.6 fold) were identified as compared to wild type embryos (Figure 6B). These data showed that the differentiation of goblet cells along the anterior-posterior axis of intestines was altered by *klf4a* knockdown.

### BrdU-Labeled S Phase and p-Histone H3-Labeled M Phase Cell Population Detected in *klf4a* Knockdown and Overexpressed Embryos

**Figure 7:**
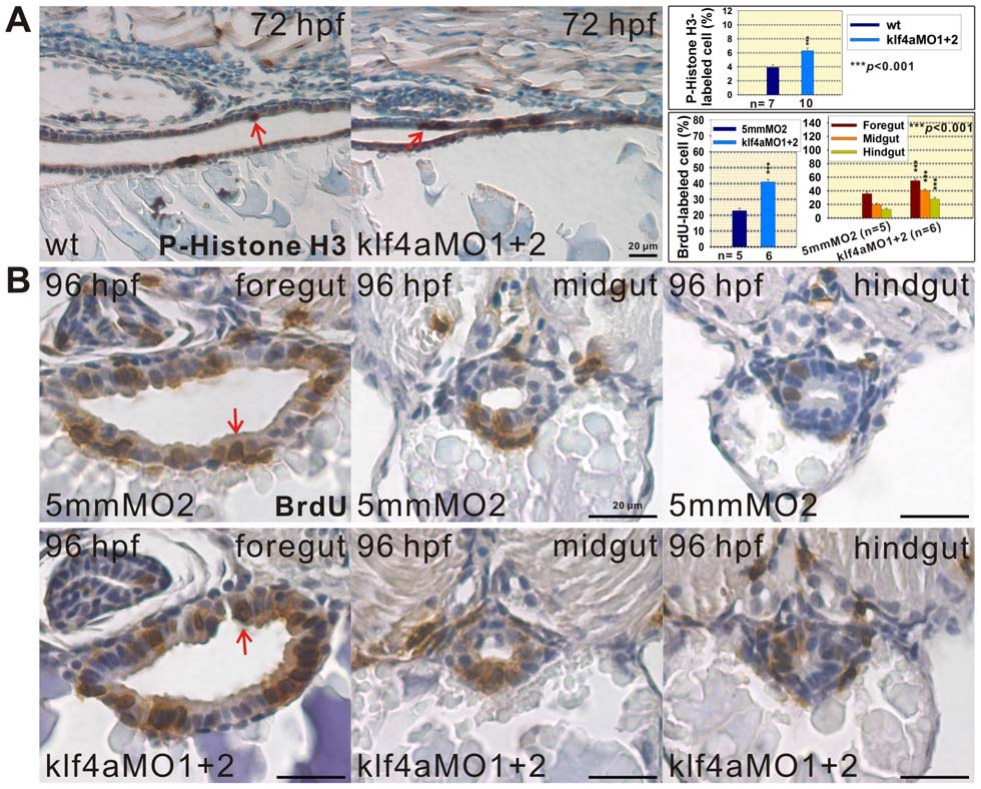
Increased cell proliferation is detected in intestines of *klf4a* morphants. (A) Images of p-Histone H3-stained cells in intestines of 72-hpf wild type and klf4a-MO1 and klf4a-MO2-injected embryos are shown. Comparison of p-Histone H3-stained cell percentages in intestines of wild type and *klf4a* morphants is shown. Arrows indicate p-Histone H3-stained cells. Error bars indicate the standard error. Student's *t*-test was conducted to compare *klf4a* morphants with wild type embryos. (B) Images of BrdU-labeled cells in the foregut, midgut, and hindgut of 96-hpf klf4a-5mmMO2-injected and klf4a-MO1 and klf4a-MO2-injected embryos are shown. Comparison of BrdU-labeled cell percentages in intestines and in respective foregut, midgut and hindgut of klf4a-5mmMO2-injected and *klf4a* morphants are shown in panel A. Arrows indicate BrdU-labeled cells. Error bars indicate the standard error. Student's *t*-test was conducted to compare *klf4a* morphants with klf4a-5mmMO2-injected embryos.

**Figure 8:**
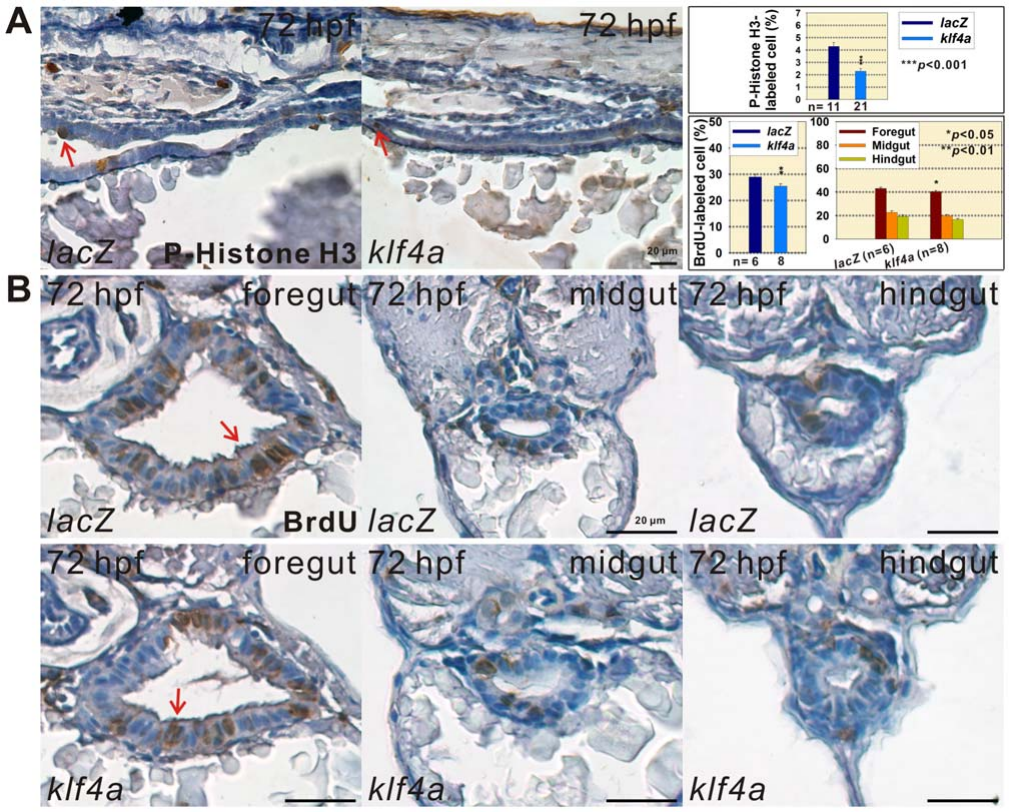
Decreased cell proliferation is detected in intestines of *klf4a*-overexpressed embryos. (A) Images of p-Histone H3-stained cells in intestines of 72-hpf *lacZ*- and *klf4a*-overexpressed embryos are shown. Comparison of p-Histone H3-stained cell percentages in intestines of *lacZ*- and *klf4a*-overexpressed embryos is shown. Arrows indicate p-Histone H3-stained cells. Error bars indicate the standard error. Student's *t*-test was conducted to compare *klf4a*- with *lacZ*-overexpressed embryos. (B) Images of BrdU-labeled cells in the foregut, midgut, and hindgut of 72-hpf *lacZ*- and *klf4a*-overexpressed embryos are shown. Comparison of BrdU-labeled cell percentages in intestines and in respective foregut, midgut, and hindgut of *lacZ*- and *klf4a*-overexpressed embryos are shown in panel A. Arrows indicate BrdU-labeled cells. Error bars indicate the standard error. Student's *t*-test was conducted to compare *klf4a*- with *lacZ*-overexpressed embryos.

To investigate the role of Klf4a in zebrafish intestinal cell proliferation, BrdU labeling and p-Histone H3 immunostaining were performed. A significant 1.6 fold (*n* = 10) increase of p-Histone H3-stained M phase cell percentages was observed in intestines of 72-hpf *klf4a* morphants as compared to wild type (*n* = 7) embryos (Figure 7A). A significant 1.8 fold increase of BrdU-labeled S phase cell percentages was detected in intestines of 96-hpf *klf4a* morphants (*n* = 6) with respective increases in the intestinal bulb/foregut (1.5 fold), mid-intestine/midgut (2.1 fold), and posterior intestine/hindgut (2.2 fold) regions as compared to control embryos (*n* = 5) (Figure 7A and 7B). In contrast, a significant 1.9 fold (*n* = 21) decrease of p-Histone H3-stained M phase cell percentages was observed in intestines of 72-hpf *klf4a* overexpressed embryos as compared to *lacZ*-ectopically expressed (*n* = 11) embryos (Figure 8A). And a significant 1.2 fold (*n* = 8) decrease of BrdU-labeled S phase cell percentages was detected in intestines of 72-hpf *klf4a*-ectopically expressed embryos (*n* = 8) with respective decreases in the intestinal bulb (1.1 fold), mid-intestine (1.1 fold), and posterior intestine (1.2 fold) regions as compared to *lacZ*-overexpressed embryos (*n* = 6) (Figure 8A and 8B). These results indicate that Klf4a negatively regulates intestinal cell proliferation in zebrafish embryos.

**Figure 9:**
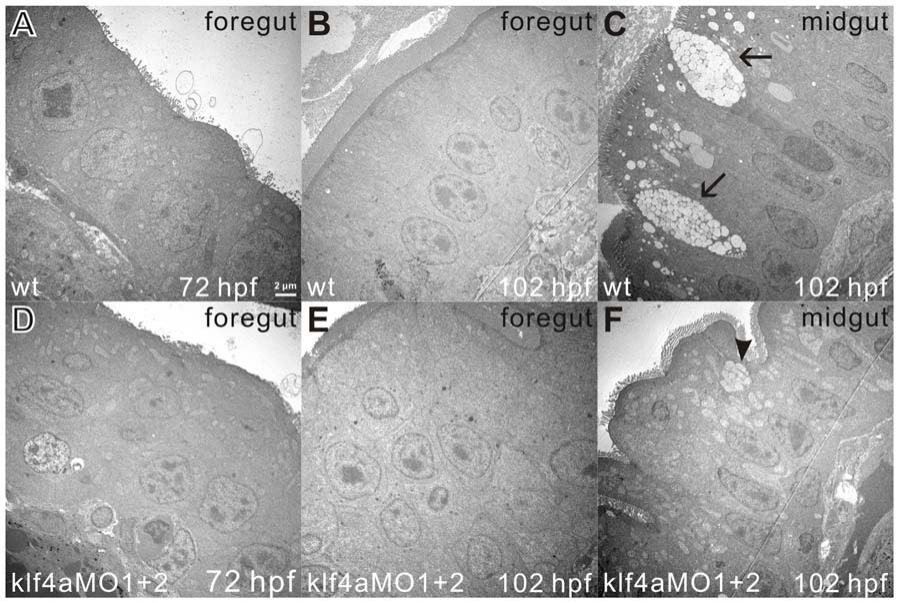
Transmission electron microscopy shows stratification of intestinal cells and abnormal goblet cell structures in *klf4a* morphants. Foregut images of a 72-hpf wild type (A) and a klf4a-MO1 and klf4a-MO2-injected (D) embryos are shown. Images of foregut (B) and midgut (C) of 102-hpf wild type and foregut (E) and midgut (F) of klf4a-MO1 and klf4a-MO2-injected embryos are shown. Arrows indicate mature goblet cells and arrowhead indicates an immature goblet cell.

In view of the fact that the presence of abnormal appearance of intestinal epithelium by histological analyses and decreased goblet cell number in *klf4a* morphants, we used transmission electron microscopy to better define the ultrastructures of enterocytes and goblet cells. A single layer of columnar shaped intestinal epithelium with basal nuclei was detected in intestinal bulbs of 72- and 102-hpf wild type embryos (Figure 9). In contrast, three layers of stratified cuboidal shaped epithelium were detected in the intestinal bulbs of 72- and 102-hpf *klf4a* morphants. In the mid-intestines of 102-hpf wild type embryos, matured goblet cells with many mucous granules which grouped into swollen thecae can be detected, while the majority of goblet cells were pre-goblet cells with a small number of mucous granules in the mid-intestines of 102-hpf *klf4a* morphants. These results further demonstrate that zebrafish Klf4a inhibits intestinal cell proliferation and regulates the differentiation of goblet cells.

### Gene Expression Profile Comparison between *klf4a* Morphants and Wild Type Embryos via DNA Microarray and Gene Ontology (GO) Analyses

To investigate the difference of gene expression profile between wild type and *klf4a* morphants, DNA microarray analyses were conducted by hybridization with Cy3-labeled double-stranded cDNAs synthesized from 96-hpf wild type and *klf4a* morphant embryos. We obtained 219 genes that are up- or down-regulated for over two-fold changes between wild type and *klf4a* morphants from DNA microarray analyses. However only 156 out of 219 genes contained gene symbols that were uploaded to the website of Gene Ontology Enrichment Analysis Software Toolkit and analyzed through Batch-Genes program using the database of *Danio rerio* with p-value smaller than 0.1. Among them, 67 genes were mapped to different GO terms (Table 1). Expression of *Stk36*, a serine/threonine kinase that stabilizing full-length Gli, was down-regulated in *klf4a* morphants [35]. This data suggests that Klf4a regulates Hedgehog signaling. Decreased expressions of *caprin2* and *tcf3b*, two mediators of canonical Wnt signaling, were identified in *klf4a* morphants. This result implies that Klf4a modulates Wnt signaling [36], [37]. In *klf4a* morphants, down-regulated expression of *cldnj* which is important for the formation of the otoliths and ear function suggests the involvement of Klf4a in the development of otic vesicle [38]. Decreased expression of *dnah9*, which is a component of outer dynein arm complex of cilia was also identified in *klf4a* morphants [39]. These results demonstrated that Klf4a may be involved in the regulation of hedgehog and Wnt signaling pathways and the development of brain, otic vesicle, and ciliagenesis.

**Table 1:** Genes up- and down-regulated in *klf4a* morphants.

Gene Ontology	Term	Down-regulated gene name	Up-regulated gene name	p-value
Biological process	smoothened signaling pathway	stk36		0.0441
	ciliary or flagellar motility	dnah9		0.0441
	biomineral tissue development	cldnj		0.0345
	positive regulation of Wnt receptor signaling pathway	caprin2		0.0345
	spinal cord dorsal/ventral patterning	TCF-3-B		0.0225
	pseudouridine synthesis	pus7l		0.05
	unsaturated fatty acid metabolic process	alox5		0.05
	lipid localization		vtg6, vtg7	0.0441
	DNA-dependent DNA replication initiation	mcm7		0.0441
	circadian rhythm	nfil3		0.0668
	response to xenobiotic stimulus		arnt	0.0904
Molecular function	peptidase activity	ctsbb, ctskl	cpa2	0.00025
	serine-type endopeptidase activity	homolog of CLIPB11 (*Anopheles gambiae*)	granzyme E, homolog of CELA1 (*Homo sapiens*)	0.0000162
	hyaluronic acid binding	hapln2		0.0441
	FMN binding		hao2	0.0904
	single-stranded DNA binding	ercc5		0.0441
Cellular component	large ribosomal subunit	rpl13a		0.0441

## Discussion

Mammalian Klf4 has been shown to play important roles in the regulation of proliferation, differentiation and tumorigenesis of gastrointestinal tract epithelium [40]. In this study, we characterized function of zebrafish *klf4a*, a zebrafish homologue of mammalian *klf4*, to gain further understanding of the role of Klf4 in the intestinal cell proliferation and differentiation. Our loss-of-function, overexpression, rescue, histological and transmission electron microscopy results demonstrated that zebrafish Klf4a is essential for the repression of intestinal cell proliferation as well as for the promotion of goblet cell differentiation and the terminal differentiation of enterocytes. In addition, zebrafish *klf4a* expression is negatively regulated by Notch signaling.

### Zebrafish Klf4a is Essential for the Differentiation of Different Intestinal Cell Lineages

Mouse *klf4* is expressed abundantly in the colon. A previous study using *Klf4* ^−/−^ mice and a recent report using *Klf4^ΔIS^* mice with intestine-specific *Klf4* deletion all showed failure of differentiation of goblet cells in the colon [19], [41]. Significant decreased mature and immature goblet cell numbers were detected in intestines of 102- and 120-hpf *klf4a* morphants (Figure 4). In addition, goblet cells were mainly distributed in the mid-intestine and their cell number was significantly affected in 102-hpf *klf4a* morphants (Figure 6). Although a small but significant decreased *glucagon*-expressing enteroendocrine cell number was also detected in intestines of 96-hpf *klf4a* morphants, nevertheless, such decline is relatively small compared to those observed for goblet cell (Figure 5). Therefore, our results demonstrate an evolutionary conserved role of Klf4 in the differentiation of goblet cell in intestinal epithelium.

Since a change in cell fate from Paneth/goblet cells to enterendocrine cells was observed in *Gfi1* ^−/−^ mice [42], we hypothesize that a portion of decreased goblet cells may change their fate in development and become enteroendocrine cells. This process results a small but significant decrease in the number of *glucagon*-expressing enteroendocrine cells observed in *klf4a* morphants. Meanwhile, the other portion of decreased goblet cells may enter apoptosis in *klf4a* morphants, which remains to be determined.

Notch signaling has been shown to be important in cell fate decision between secretory versus absorptive intestinal cells [34]. Our results show that Notch signaling inhibits *klf4a* expression in the intestine of zebrafish embryos (Figure 1), this finding is consistent with previous reports that *klf4* expression is prevented by the Notch signaling in mouse intestinal epithelium [21], [43]. Since the absence of Notch activation favors the differentiation of secretory cells in zebrafish intestine [34], we detected decreased goblet cell number in *NICD*-ectopically expressed 102-hpf embryos (Figure 4E). However, we were able to detect recovery of goblet cell number in 102-hpf embryos that had been co-injected with both *klf4a* and *Notch 1a NICD* mRNA (Figure 4E). These results further demonstrate that the regulation of differentiation of goblet cells in zebrafish intestine by Notch signaling at least partially mediated through Klf4a.

*Math1* is required for the commitment of different secretory cell lineages in mouse intestine and its expression is inhibited by Hes1 mediator of Notch signaling [44]. Although orthologue of the *Math1* has not been identified in zebrafish, we consider that zebrafish Klf4a may situate downstream of *Math1* orthologue to regulate the differentiation of goblet cells in zebrafish embryos.

It seems that the prevention of mouse *Klf4* expression by Notch signaling favors the formation of intestinal enterocytes, however, studies have shown that Klf4 regulates expression of several terminal differentiation marker genes of enterocytes [17], [18]. In addition, a recent report showed that a reduction in alkaline phosphatase activity in enterocytes of small intestine and a reduction of carbonic anhydrase-1 expression in colonocytes of *Klf4^ΔIS^* mice [41]. Likewise, decreased *PepT1* expression observed in *klf4a* morphants indicates that Klf4a is required for the terminal differentiation of enterocytes in zebrafish embryos (Figure 3).

### Zebrafish Klf4a Represses Intestinal Cell Proliferation

Although low *Klf4* expression levels were identified in different human colorectal cancer cell lines, there are controversial results regarding *in vivo* Klf4 functions as a tumor suppressor from different mouse studies [11], [19], [20], [41]. In *klf4a* morphants, increased intestinal cell proliferation and the presence of stratified enterocytes were detected while decreased intestinal cell proliferation was identified in *klf4a*-ectopically expressed embryos (Figure 7, 8 and 9). We consider that substantial decreased *PepT1* expression level was not detected in 120-hpf *klf4a* morphants is likely attributed to increased layers of enterocytes with low level of *PepT1* expression. Mammalian Klf4 was shown to mediate the cell cycle progression by repress expressions of *cyclin D1* and *cyclin B1* either directly or indirectly through the activation of *p21* [45]. In addition, Klf4 was found to interact with β-catenin and repress β-catenin-mediated gene expression such as *cyclin D1* [22]. Recent studies in zebrafish embryos showed that expression of *p21* and *cyclin G1* inhibited cell proliferation in the liver, intestine and exocrine pancreas [46], [47]. Furthermore, Wnt-β-catenin signaling was shown to maintain cell proliferation in intestines of zebrafish embryos [48]. Therefore, we consider that Klf4a may regulate expressions of *p21*, *cyclin G1* and/or β-catenin-mediated genes to affect intestinal cell proliferation in zebrafish embryos.

### Zebrafish Klf4a Regulates the Development of Brain, Otic Vesicle and Ciliagenesis

In 24-hpf *klf4a* morphants, notable morphological changes including smaller eyes and otic vesicles, reduced dorsal-ventral axis, and the enlargement of midbrain ventricle were detected (Figure 2). Results of DNA microarray and GO analyses revealed decreased expressions of *caprin2* and *tcf3b* in *klf4a* morphants (Table 1) [37]. Furthermore, elevated expression level of *vim* was observed in the hindbrain of 24-hpf *klf4a* morphants (unpublished observation). All these results imply that Klf4a is involved in the brain development.

Decreased expression level of *cldnj* that was shown to be essential for normal ear function in zebrafish, and small otic vesicles with the presence of small otoliths were identified in *klf4a* morphants (Table 1) [38]. These data suggest that Klf4a is required for the proper development of otic vesicles. DNA microarray and GO analyses showed reduced expression levels of *stk36* and *dnah9* in *klf4a* morphants (Table 1). Since zebrafish Stk36 was demonstrated to be required for cilia biogenesis [49], we hypothesize that Klf4a is also involved in the regulation of ciliagenesis.

Using zebrafish embryos, our results provide *in vivo* evidence that zebrafish Klf4a functions to inhibit cell proliferation, and confirm its role as a tumor suppressor. Together with observation in human and mouse, Klf4a and its homologues are proved essential for the differentiation of goblet cells and the terminal differentiation of enterocytes in zebrafish embryos. Furthermore, *klf4a* expression is negatively regulated by Notch signaling and Klf4a acts as a mediator in the regulation of differentiation of goblet cells in zebrafish intestine by Notch signaling.

## Supporting Information

Figure S1 **Amino acid sequence comparison and phylogenetic analyses.** (A) Amino acid sequence comparison among human and mouse Klf4 and zebrafish Klf4a. Identical amino acid sequences are shown in black boxes. Conserved acidic residues important for activation are indicated by star. Three zinc finger DNA binding motifs are underlined. (B) Phylogenetic tree analyses. The bar represents the number of estimated differences for a unit branch length. Bootstrap values are shown.
(DOC)

Figure S2 **Maps showing gene order with conserved syntenic groups between zebrafish linkage group 21 and respective human chromosome 9 and mouse chromosome 4.** Genes and their start positions (million base pair) on respective zebrafish linkage group 21 (LG 21), human chromosome 9 (Has 9) and mouse chromosome 4 (Mmu 4) are shown. The entire chromosome (grey box) is shown, but the scale is different in different chromosomes.
(DOC)

Figure S3 **Developmental expression patterns of** ***klf4a*** **.** Lateral view of 48- (A), 72- (B), and 96-hpf (C), and dorsal view of 96-hpf embryos (D), and ventral view of 72- (E) and 96-hpf (F) deyolked embryos are shown. CN, cranial neuron; E, epidermis; EP, exocrine pancreas; HN, habenular neuron; I, intestine; L, liver; OB, olfactory bulbs; P, pharynx; PA, pharyngeal arches; PF, pectoral fin; R, retina.
(TIF)

Figure S4 **Phenotype comparison among 4 ng klf4a-MO1-injected and 16 ng klf4a-MO2-injected morphants and 4 ng klf4a-5mmMO1-injected embryos.** (A) Phenotype of 24-hpf embryos with different treatments. * Indicates enlargement of the midbrain ventricles and arrows indicate reduced otic vesicles. (B) Intestinal phenotype of 96-hpf klf4a-MO1-injected and klf4a MO2-injected morphants. Arrows indicate the absence of intestinal folding and arrowheads indicate no inflation of swim bladder.
(TIF)

Table S1 **Morphant phenotype characterization based on injection of different doses of** ***klf4a*** **MO1 and** ***klf4a*** **MO2.**
(DOC)
