# Supplementary material for: Zebrafish Krüppel-Like Factor 4a Represses Intestinal Cell Proliferation and Promotes Differentiation of Intestinal Cell Lineages
Source: PLoS One. 2011 Jun 8;6(6):e20974. doi: 10.1371/journal.pone.0020974 (PMC3110806; doi:10.1371/journal.pone.0020974)
Supplement: Figure S1 — Amino acid sequence comparison and phylogenetic analyses. (A) Amino acid sequence comparison among human and mouse Klf4 and zebrafish Klf4a. Identical amino acid sequences are shown in black boxes. Conserved acidic residues important for activation are indicated by star. Three zinc finger DNA binding motifs are underlined. (B) Phylogenetic tree analyses. The bar represents the number of estimated differences for a unit branch length. Bootstrap values are shown. (DOC) [file pone.0020974.s001.doc]

**A**

HKlf4 **MRQPPGESD.** **MAVSDALLPS** **FSTFASGPAG** **REKTLRQAGA** **PNNRWREELS** **HMKRLPPVLP** **GRPYDLAAAT** **VATDLESGGA** **GAACGGSN.L** 88

MKlf4 **MRQPPGESD.** **MAVSDALLPS** **FSTFASGPAG** **REKTLRPAGA** **PTNRWREELS** **HMKRLPP.LP** **GRPYDL.AAT** **VATDLESGGA** **GAACSSNN.P** 86

**ZKlf4a** **MRQPPTEFDS** **MALSGTILPS** **ISTFASGTDV** **KHKAVLGPGA** **AGSRWKEEMS** **HLKR..PCM.** **..........** **........ST** **GATCADQDVP** 69

HKlf4 **APLPRRETEE** **FNDLLDLDFI** **LSNSLTHPPE** **SVAATVSSSA** **SASSSSSPSS** **SGPASAPSTC** **SFTYPIRAGN** **DPGVAPGGTG** **GGLLYGRESA** 178

MKlf4 **ALLARRETEE** **FNDLLDLDFI** **LSNSLTH.QE** **SVAATVTTSA** **SASSSSSPAS** **SGPASAPSTC** **SFSYPIRAGG** **DPGVAASNTG** **GGLLYSRESA** 175 **ZKlf4a** **PVMAKIEPEE** **L.ELLDYDFI** **LSNSLLQQQQ** **QQQASTSRTI** **..........** **..PASSPTHY** **TFPMP.....** **....SPQGQE** **GSMLY.....** 132

*** ** * ***

HKlf4 **PPPTAPFNLA** **DINDVSPSGG** **FVAELLRPEL** **DPVYIPPQQP** **QPPGGGLMGK** **FVLKASLSAP** **GSEYGSPSVI** **SVSKGSPDGS** **HPVVVAPYNG** 268

MKlf4 **PPPTAPFNLA** **DINDVSPSGG** **FVAELLRPEL** **DPVYIPPQQP** **QPPGGGLMGK** **FVLKASLTTP** **GSEYSSPSVI** **SVSKGSPDGS** **HPVVVAPYSG** 265

**ZKlf4a** **...TIP....** **DISDVSPSGG** **FVAELMRPEL** **DPAYLQPT..** **.....SLHGK** **FVVKTTMDM.** **.SDYNQG..V** **SISK...DAS** **..........** 191

HKlf4 **GPPRTCPKIK** **QEAVSSCT..** **.....HLGAG** **PPLSNGHRPA** **...AHDFPLG** **RQLPSRTTPT** **LGLEEVLSSR** **DCHPALP.LP** **PGFHPHPGPN** 347

MKlf4 **GPPRMCPKIK** **QEAVPSCTVS** **RSLEAHLSAG** **PQLSNGHRPN** **...THDFPLG** **RQLPTRTTPT** **LSPEELLNSR** **DCHPGLP.LP** **PGFHPHPGPN** 351

**ZKlf4a** **AVPFACPRIK** **QECPSTCTAS** **RPMDLHLRGS** **SAQSGVHQGS** **MLDPHAFSSG** **RGARSSLSP.** **..........** **DEHPQAPGLG** **SGYHPNTA..** 268

HKlf4 **YPSFLPDQMQ** **PQVPPLHYQE** **LMPPGSCMPE** **EPKPKRGRRS** **WPRKRTATHT** **CDYAGCGKTY** **TKSSHLKAHL** **RTHTGEKPYH** **CDWDGCGWKF** 437

MKlf4 **YPPFLPDQMQ** **SQVPSLHYQE** **LMPPGSCLPE** **EPKPKRGRRS** **WPRKRTATHT** **CDYAGCGKTY** **TKSSHLKAHL** **RTHTGEKPYH** **CDWDGCGWKF** 441

**ZKlf4a** **YSGF.P...Q** **APAQSLQYQE** **LISPAEGLPE** **ESKPKRGRRS** **WPRKRIATHT** **CDYAGCGKTY** **TKSSHLKAHH** **RTHTGEKPYH** **CDWEGCGWKF** 354

**Similarity(%)**

HKlf4 **ARSDELTRHY** **RKHTGHRPFQ** **CQKCDRAFSR** **SDHLALHMKR** **HF** 479 66.1%

MKlf4 **ARSDELTRHY** **RKHTGHRPFQ** **CQKCDRAFSR** **SDHLALHMKR** **HF** 483 67.7%

**ZKlf4a** **ARSDELTRHY** **RKHTGIRPFQ** **CLKCDRAFSR** **SDHLALHMKR** **HL** 396 100.0%

**B**
