# Supplementary figures and images for: Zebrafish Krüppel-Like Factor 4a Represses Intestinal Cell Proliferation and Promotes Differentiation of Intestinal Cell Lineages
Source: PLoS One. 2011 Jun 8;6(6):e20974. doi: 10.1371/journal.pone.0020974 (PMC3110806; doi:10.1371/journal.pone.0020974)

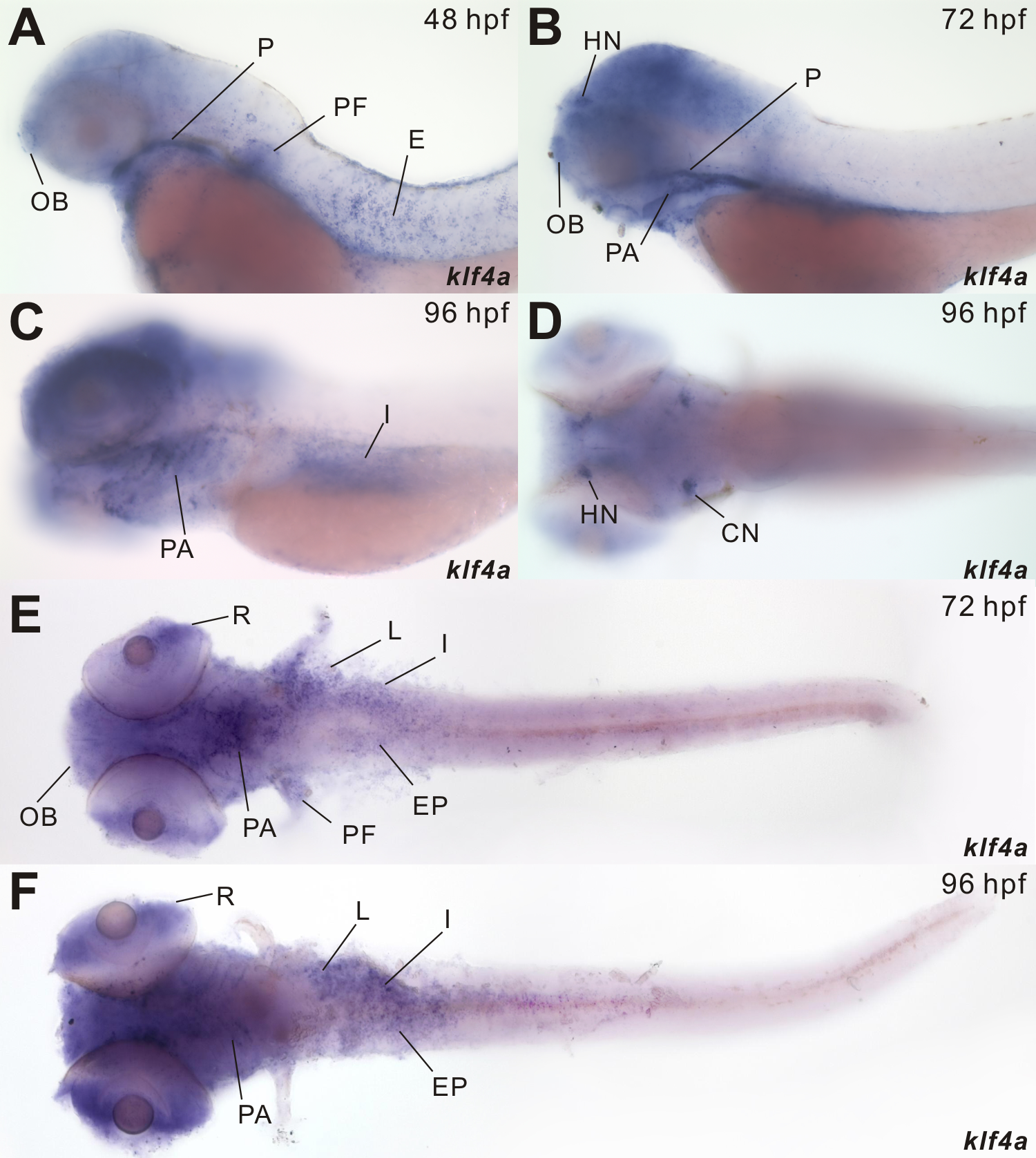

Supplement: Figure S3 — Developmental expression patterns of klf4a . Lateral view of 48- (A), 72- (B), and 96-hpf (C), and dorsal view of 96-hpf embryos (D), and ventral view of 72- (E) and 96-hpf (F) deyolked embryos are shown. CN, cranial neuron; E, epidermis; EP, exocrine pancreas; HN, habenular neuron; I, intestine; L, liver; OB, olfactory bulbs; P, pharynx; PA, pharyngeal arches; PF, pectoral fin; R, retina. (TIF) [file pone.0020974.s003.tif]

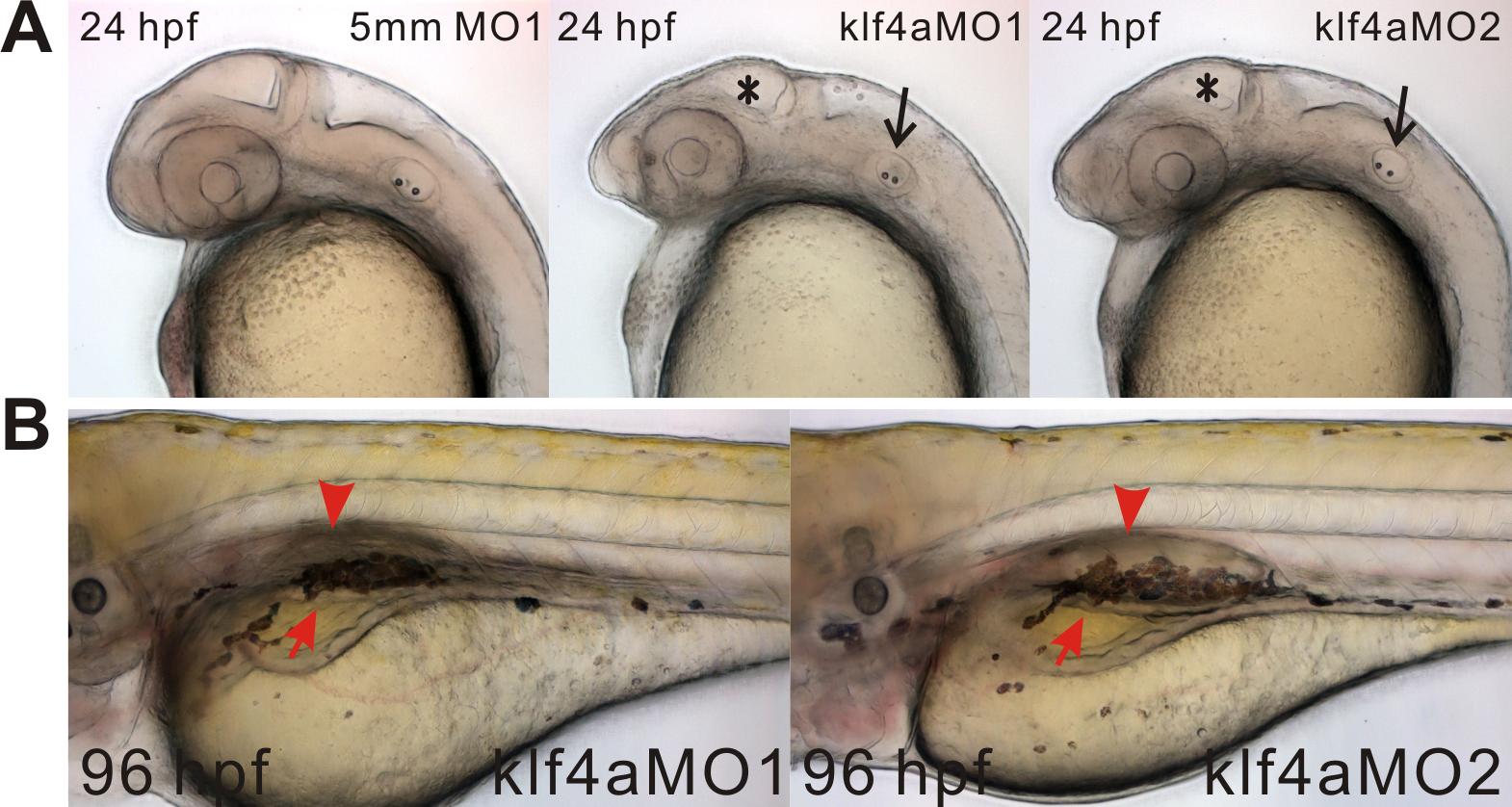

Supplement: Figure S4 — Phenotype comparison among 4 ng klf4a-MO1-injected and 16 ng klf4a-MO2-injected morphants and 4 ng klf4a-5mmMO1-injected embryos. (A) Phenotype of 24-hpf embryos with different treatments. * Indicates enlargement of the midbrain ventricles and arrows indicate reduced otic vesicles. (B) Intestinal phenotype of 96-hpf klf4a-MO1-injected and klf4a MO2-injected morphants. Arrows indicate the absence of intestinal folding and arrowheads indicate no inflation of swim bladder. (TIF) [file pone.0020974.s004.tif]
