# Supplementary material for: Zebrafish Krüppel-Like Factor 4a Represses Intestinal Cell Proliferation and Promotes Differentiation of Intestinal Cell Lineages
Source: PLoS One. 2011 Jun 8;6(6):e20974. doi: 10.1371/journal.pone.0020974 (PMC3110806; doi:10.1371/journal.pone.0020974)
Supplement: Table S1 — Morphant phenotype characterization based on injection of different doses of klf4a MO1 and klf4a MO2. (DOC) [file pone.0020974.s005.doc]

| **Injected amount** | **Wild type (%)** | **Morphant (%)** | **Death (%)** | **Deformity (%)** | **Total (*n*)** |
| --- | --- | --- | --- | --- | --- |
| MO1 (4 ng) | 6.5±0.06 | 91.7±0.06 | 0.9±0.02 | 0.9±0.02 | 442 |
| MO1 (8 ng) | 1.6±0.03 | 80.5±0.09 | 2.9±0.04 | 14.9±0.08 | 429 |
| MO2 (8 ng) | 89.5±0.06 | 9.3±0.06 | 1±0.02 | 0.2±0.009 | 489 |
| MO2 (16 ng) | 34.3±0.1 | 61.9±0.2 | 3.8±0.06 | 0 | 306 |
| MO1+2 (1.5 ng+1.5 ng) | 0 | 98.1±0.02 | 0.9±0.02 | 1±0.02 | 580 |

**Table S1. Morphant phenotype characterization based on injection of different doses of *klf4a* MO1 and *klf4a* MO2**
