# Supplementary material for: Zebrafish Krüppel-Like Factor 4a Represses Intestinal Cell Proliferation and Promotes Differentiation of Intestinal Cell Lineages
Source: PLoS One. 2011 Jun 8;6(6):e20974. doi: 10.1371/journal.pone.0020974 (PMC3110806; doi:10.1371/journal.pone.0020974)
Supplement: Supplementary file 4 [file pone.0020974.s002.doc]

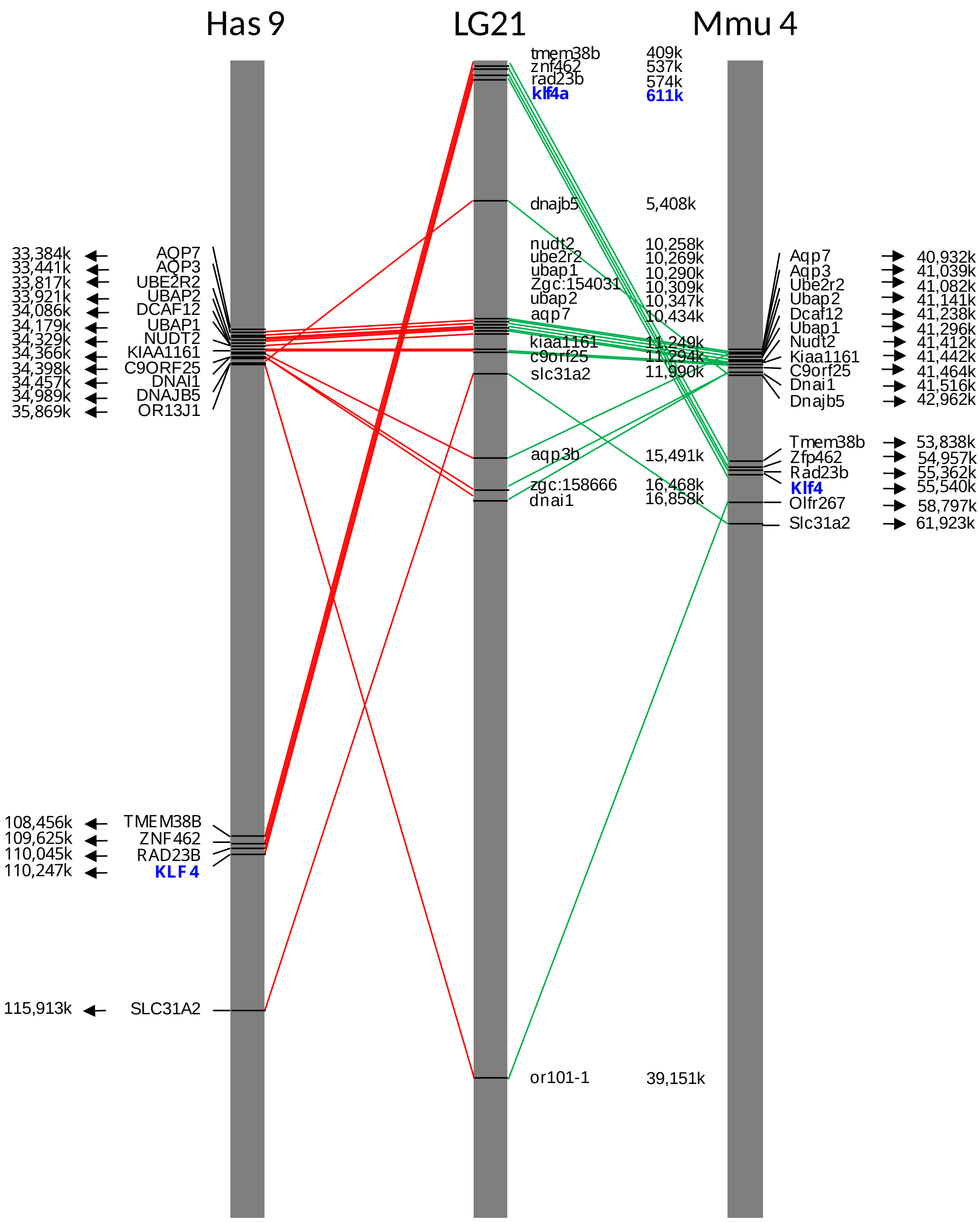

Has 9
LG21
Mmu 4
33,384k AQP7
33,441k AQP3
33,817k UBE2R2
33,921k UBAP2
34,086k DCAF12
34,179k UBAP1
34,329k NUDT2
34,366k KIAA1161
34,398k C9ORF25
34,457k DNAI1
34,989k DNAJB5
35,869k OR13J1
108,456k TMEM38B
109,625k ZNF462
110,045k RAD23B
110,247k KLF4
115,913k SLC31A2
tmem38b 409k
znf462 537k
rad23b 574k
klf4a 611k
dnajb5 5,408k
nudt2 10,258k
ube2r2 10,269k
ubap1 10,290k
Zgc:154031 10,309k
ubap2 10,347k
aqp7 10,434k
kiaa1161 11,249k
c9orf25 11,294k
slc31a2 11,990k
aqp3b 15,491k
zgc:158666 16,468k
dnai1 16,858k
or101-1 39,151k
Aqp7 40,932k
Aqp3 41,039k
Ube2r2 41,082k
Ubap2 41,141k
Dcaf12 41,238k
Ubap1 41,296k
Nudt2 41,412k
Kiaa1161 41,442k
C9orf25 41,464k
Dnai1 41,516k
Dnajb5 42,962k
Tmem38b 53,838k
Zfp462 54,957k
Rad23b 55,362k
Klf4 55,540k
Olfr267 58,797k
Slc31a2 61,923k
